# Identification of biallelic loss-of-function PREP variants in three individuals with syndromic intellectual disability

**DOI:** 10.1136/jmg-2026-111501

**Published:** 2026-06-26

**Authors:** Erik Hertstein, Miriam Bertrand, Johannes Kopp, Henrike Lisa Sczakiel, Oliver Küchler, Nicolai von Kügelgen, Björn Fischer-Zirnsak, Gabriele Hildebrand, Jonas Leubner, Denise Horn, Stefan Mundlos, Tobias B Haack, Angela Kaindl, Christoph G Korenke, Felix Boschann

**Affiliations:** 1 Institute of Medical Genetics and Human Genetics, Charité – Universitätsmedizin Berlin, Corporate Member of Freie Universität Berlin and Humboldt-Universität zu Berlin, Berlin, Germany; 2 Institute of Medical Genetics and Applied Genomics, University of Tübingen, Tübingen, Germany; 3 BIH Biomedical Innovation Academy, BIH Charité Clinician Scientist Program, Berlin Institute of Health at Charité – Universitätsmedizin Berlin, Berlin, Germany; 4 Exploratory Diagnostic Sciences (EDS), Berlin Institute of Health at Charité – Universitätsmedizin Berlin, Berlin, Germany; 5 Core Unit Bioinformatics (CUBI), Berlin Institute of Health at Charité – Universitätsmedizin Berlin, Berlin, Germany; 6 German Center for Child and Adolescent Health (DZKJ), Berlin, Germany; 7 Department of Pediatric Neurology, Charité – Universitätsmedizin Berlin, Corporate Member of Freie Universität Berlin and Humboldt-Universität zu Berlin, Berlin, Germany; 8 Center for Rare Diseases, University Hospital Tübingen, Tübingen, Germany; 9 Department of Neuropediatrics, Children’s Hospital, Klinikum Oldenburg, Oldenburg, Germany

**Keywords:** Epilepsy, Genomics, RNA-Seq, RNA Splicing, Mental Disorders

## Abstract

**Background:**

Neurodevelopmental disorders are one of the most prevalent reasons for genetic testing in childhood. Despite the identification of over 1950 associated genes, many proposed candidate genes lack convincing gene-disease validity. The gene *PREP* encodes the broadly expressed prolyl endopeptidase whose exact function remains largely unknown. A homozygous *PREP* variant has been reported once as a candidate gene in two siblings with intellectual disability but no functional studies were conducted.

**Methods:**

Exome and trio genome sequencing were performed in two unrelated families as part of larger cohorts. Segregation analysis, RNA sequencing and immunoblots were performed to further examine the pathogenicity of detected *PREP* variants.

**Results:**

We report three individuals from two unrelated families who presented with intellectual disability, behavioural abnormalities, strabismus, generalised muscular hypotonia, dysmorphic facial features and epilepsy. Exome and genome sequencing identified two different homozygous rare *PREP* variants: c.1570_1573dup, p.(Asn525Thrfs*5) and c.1839-2A>G, p.?. RNA sequencing confirmed the detected intronic variant to result in two aberrant mRNA isoforms. In patient-derived cells immunoblots showed absence of PREP protein.

**Conclusion:**

Our data suggest PREP deficiency as the underlying cause of a syndromic neurodevelopmental disorder.

**WHAT IS ALREADY KNOWN ON THIS TOPIC**

- A homozygous variant in *PREP* has been reported in two siblings in the supplemental information of a large intellectual disability/neurodevelopmental disorder cohort study but the evidence was limited to support a causal role.

**WHAT THIS STUDY ADDS**

- We demonstrate that biallelic loss-of-function variants in *PREP* are the likely underlying cause of a syndromic neurodevelopmental disorder.

**HOW THIS STUDY MIGHT AFFECT RESEARCH, PRACTICE OR POLICY**

- The *PREP* gene should be considered in patients with intellectual disability or syndromic epilepsy and added to virtual panels.

## Introduction

 Intellectual disability (ID) is characterised by deficits in intellectual and adaptive functioning with onset during childhood and represents a subtype within the broader group of neurodevelopmental disorders (NDD).^1^ ID affects a substantial number of children worldwide with varying estimation of global prevalence depending on applied definition and methodology. A systematic review from 2022 using the Diagnostic and Statistical Manual of Mental Disorders, Fifth Edition criteria estimated the prevalence at 0.63%, whereas the 2019 Global Burden of Disease data suggested a prevalence of around 2% among children.^2 3^ Besides various other non-genetic causes, >1950 genes have been related to ID/NDD according to the SysNDD database.^4^ Due to the vast genetic heterogeneity, exome and genome sequencing (ES, GS) are recommended as first-line diagnostic tests.^5^ However, the diagnostic yield often remains below 50%.^6 7^ One explanation is the high number of candidate genes with insufficient supporting evidence.^8^ In this regard, the SysNDD database currently lists 750 genes with limited evidence specifically for ID.^4^ One of these genes is *PREP* (HGNC:9358, SysNDD identifier: 2382). The annotation is based on supplementary information from a publication in which a homozygous frameshift variant in *PREP* was detected in two siblings with moderate ID.^9^ Interestingly, *Prep*-knockout (KO) mice also exhibit a neurological phenotype with overactivity, anxiety, disturbed synaptic plasticity and reduced brain volume.^10^

*PREP* encodes the enzyme prolyl endopeptidase (PREP) (synonym: prolyl oligopeptidase, EC 3.4.21.26), which belongs to the serine S9 peptidase family.^11^ PREP is detectable in almost all tissues and expressed during neuronal differentiation and in mature human neurons.^12^ It consists of 710 amino acids (aa) and has a molecular weight of approximately 81 kDa. The enzyme comprises a classic α-β hydrolase protease domain formed by aa residues 1–72 and 428–710 (catalytic triad: Ser554, Asp641, His680), and a non-catalytic domain (aa residues 73–427) with a seven-bladed β-propeller shape.^13 14^ The two domains are connected by short linker hinges.^14^ PREP specifically cleaves oligopeptide substrates at peptide bonds C-terminal to proline residues via the protease domain.^13^ Furthermore, PREP has been proposed to be implicated in molecular processes via protein-protein interactions (PPI), modulated by the β-propeller domain.^15^ However, there is no consensus regarding its substrates and interaction partners.

In this study, we report three additional individuals from two unrelated families with homozygous loss-of-function (LoF) variants and propose PREP deficiency as the underlying cause for a syndromic ID/NDD.

## Methods

### Next-generation sequencing and identification of other cases

Peripheral blood samples of patient 1 and his unaffected parents were obtained for subsequent paired-end short-read GS on an Illumina NovaSeq 6000 device at Labor Berlin, Germany. Library preparation was performed using the Twist Library Preparation EF Kit with amplification from fragmented genomic DNA. The generated read sequences were analysed using the Illumina DRAGEN Bio-IT platform (V.3.10). Variants were filtered according to allele frequency, inheritance and impact using the VarFish software.^16^ Due to known consanguinity, rare homozygous variants were prioritised. Only one candidate variant in *PREP* was convincing and further segregated in one affected sibling (patient 2) and five unaffected siblings using Sanger sequencing. Detailed results of the segregation analysis (ie, heterozygous carrier vs homozygous wildtype) for non-affected minor siblings are not presented due to privacy concerns.

By personal communication and re-analysis of ES data, we identified patient 3 from an unrelated family (family B). Diagnostic ES was performed on genomic DNA extracted from peripheral blood leucocytes as previously described.^17 18^ Briefly, coding genomic regions were enriched using the SureSelect XT Human All Exon V7 Kit (Agilent Technologies, Santa Clara, California, USA) and sequenced as 2×100 bp paired-end reads on a NovaSeq 6000 system (Illumina, San Diego, California, USA) to an average coverage of 140×. Sequence data were analysed using the megSAP pipeline.^19^ Intronic variants were assessed using SpliceAI and Pangolin scores.^20 21^

A SysNDD database query revealed two siblings from one additional family, while a GeneMatcher submission returned no matches.^9 22^

### Cell culture

Heparin blood samples of patients 1 and 3 were obtained. For the generation of immortalised lymphoblastoid cell lines (LCLs), B cells were isolated and transfected with Epstein-Barr virus EBV. Both patient LCLs and two non-related controls LCLs were cultured at 37°C and 5% CO_2_ in RPMI-1640 with L-glutamine supplemented with 10% fetal calf serum and penicillin/streptomycin (all purchased from Gibco, Thermo Fisher Scientific, Waltham, Massachusetts, USA).

### RNA-sequencing

Peripheral blood of patient 3 was collected in a PAXgene RNA tube (Qiagen, Venlo, the Netherlands) and was used for RNA-sequencing as previously described.^23^ In brief, RNA was isolated from PAXgene Blood RNA Tubes using the QIAsymphony RNA Kit (Qiagen) and enriched by poly(A) capture with the NEBNext Poly(A) mRNA Magnetic Isolation Module (New England Biolabs, Ipswich, Massachusetts, USA). Libraries were prepared using the NEBNext Ultra II Directional RNA Library Prep Kit for Illumina and the NEBNext Globin & rRNA Depletion Kit (both from New England Biolabs), following the manufacturer’s instructions. Sequencing was carried out as 2×100 bp paired-end reads on an Illumina NovaSeq X Plus system (Illumina). Splicing and expression analyses at the gene and exon levels were performed using the megSAP pipeline in comparison with an in-house cohort of 495 individuals with diverse phenotypes, analysed from the same tissue using the same processing system.^24^ Variants of interest were visually inspected with the Integrative Genomics Viewer (Broad Institute, Cambridge, Massachusetts, USA).

For complementation and confirmation, RNA was also extracted from the LCL cultures of patients 1 and 3 and transcriptome analysis was conducted as previously described.^25^

### Quantitative reverse transcriptase PCR

RNA was extracted from LCL cell pellets using the phenol/chloroform method and its integrity was confirmed with an agarose gel electrophoresis. RNA concentration was measured with a Qubit V.2.0 Fluorometer (Thermo Fisher Scientific) and transcription into cDNA was performed using the RevertAid H Minus First Strand cDNA Synthesis Kit (Thermo Fisher Scientific). HOT FIREPol EvaGreen qPCR Mix Plus (ROX) Kit (Solis BioDyne, Tartu, Estonia) was used for qRT-PCR according to the manufacturer’s recommended protocol on a QuantStudio 3 real-time PCR system (Thermo Fisher Scientific). Two different primer pairs located in *PREP* exons 9–10 and exon 15, respectively, were used. For verification of the aberrant splicing in patient 3, two additional primer pairs were used, one spanning the exon truncation site and the other spanning the intron retention site (schematic illustration of primer localisation in online supplemental material). Technical triplicates were included for each sample and three independent runs were performed. Relative expression levels were calculated with the ΔΔCt method normalised to *GAPDH*, using Microsoft Excel (Microsoft, Redmond, Washington, USA). Primer sequences are listed in online supplemental table 1.

### Immunoblot analysis

Whole cell protein lysates were obtained from flash frozen LCL cell pellets using radioimmunoprecipitation assay (RIPA) buffer (150 mM NaCl, 50 mM Tris, 5 mM EDTA, 1% Triton X-100, 0.25% desoxycholate, 5% sodium dodecyl sulfate (SDS)) containing protease inhibitor (cOmplete, Roche, Basel, Switzerland). Protein concentrations were measured using the bicinchoninic acid assay (BCA) protein assay kit (Thermo Fisher Scientific). 25 µg of total protein per sample was applied, separated using SDS-PAGE and transferred to nitrocellulose membrane (Thermo Fisher Scientific). Immunoblot detection was performed using primary antibodies against PREP (ab58988/rabbit, ab246978/rabbit, both from Abcam, Cambridge, UK). For secondary antibody detection, membranes were stained with IRDye-conjugated secondary antibodies (926-32211/800CW goat antirabbit, 926-68073/680RD donkey antirabbit, both from LI-COR Biosciences, Lincoln, Nebraska, USA). The signal detection was conducted with the OdysseyFc Imaging System and Image Studio (LI-COR Biosciences). Beta-actin (#4790/rabbit, Cell Signaling Technology, Danvers, Massachusetts, USA) was used as a loading control. The experiments were performed three times.

## Results

### Clinical reports

#### Family A

Patient 1 (male) was born at 40 weeks of gestation as the fourth child of consanguineous parents from Turkey. Postnatal adaptation was unremarkable, and birth measurements were normal: weight 3700 g (+0.17 SD), length 51 cm (−0.65 SD), occipitofrontal head circumference 36.5 cm (+0.69 SD). Generalised muscular hypotonia and delayed motor development became apparent in the first months of life. He was able to sit without support at 20 months and walked unaided at 3 years of age. A left inguinal hernia was surgically corrected. At 4 years of age, he began to speak single words. Physical examination revealed body measurements within the norm. He had bilateral pes cavus, esotropia and amblyopia of the left eye, bilateral hyperopia and astigmatism. An IQ test (Snijders-Oomen Non-verbal Intelligence Test, SON-R 2½−7) revealed moderate ID (score <50). Electroneurography showed reduced nerve conduction velocity, indicative of a demyelinating neuropathy. At the age of 10 years, the boy experienced his first focal to bilateral tonic-clonic seizure. He was initially misdiagnosed as having SeLECTS (self-limited epilepsy with centrotemporal spikes, formerly known as Rolandic epilepsy) and therefore treated with sultiam. Reassessment of the case revealed a developmental and epileptic encephalopathy (DEE). On EEG, he had background slowing and multifocal epileptiform discharges with generalisation and strong activation during sleep, but no continuous spike-wave during sleep. A cranial MRI scan revealed enlarged external cerebrospinal fluid spaces alongside age-appropriate gyration and myelination.

**Figure 1:**
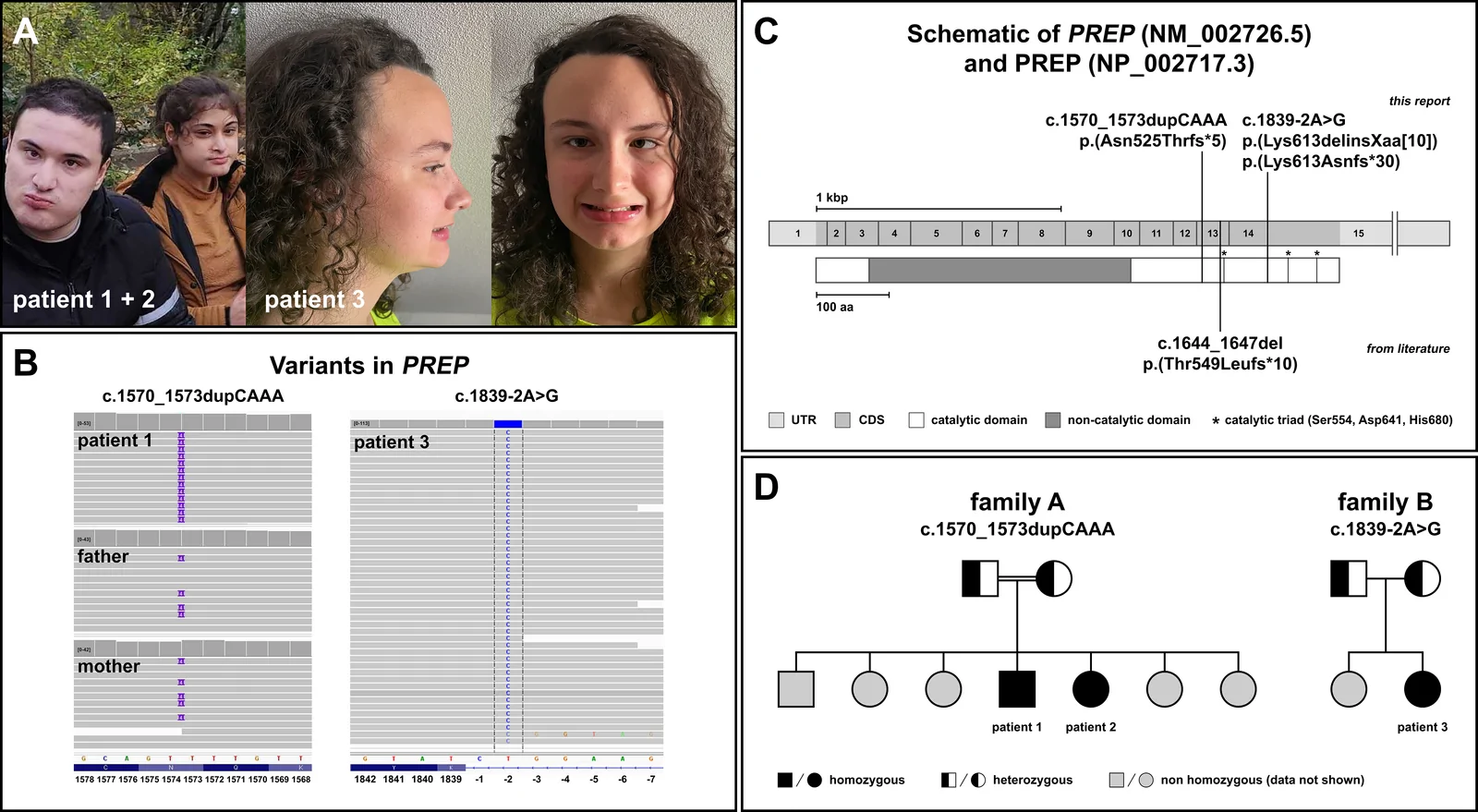
(A) Facial photographs of affected patients showing a comparable facial gestalt comprising a high hairline, upslanted palpebral fissures, a low-hanging columella and a thin upper lip. (B) Integrative Genomics Viewer screenshots showing *PREP* variants identified via trio genome sequencing in family A and single exome sequencing in family B. (C) Localisation of *PREP* variants from this report and the literature; p.(Lys613delinsXaa[10]) represents p.(Lys613delinsAsnLeuThrLysValPheLeuProSerGly). (D) Pedigree and segregation pattern of identified *PREP* variants in family A and B. aa, amino acid; CDS, coding sequence; UTR, untranslated region.

At the last clinical examination at 18 years of age, he was largely non-verbal and used a communication aid. He did not eat independently, was not toilet trained and was incontinent. The boy exhibited behavioural problems with repetitive movements, auto-aggression and aggression towards others. The following physical measurements were taken during the last examination: head circumference 60 cm (+1.98 SD), length 178 cm (−0.4 SD), weight 91 kg (+ 1.67 SD). Facial dysmorphism included a prominent forehead with a high frontal hairline, upward slanting palpebral fissures, strabismus, long columella with a short appearing philtrum and thin upper lip (figure 1A). Genetic testing (chromosomes, array and *FMR1* repeat analysis) revealed no abnormalities. He was therefore included in a GS study for unsolved cases alongside his parents.

His parents had five other healthy children and one affected daughter (patient 2) with a comparable phenotype and similar facial gestalt (figure 1A). She was born at 39+5 weeks of gestation with normal weight (3295 g, +1.16 SD), length (48 cm, −1.6 SD) and occipitofrontal head circumference (34 cm, −0.63 SD). Her psychomotoric development was delayed. At 2 years and 4 months, she began to walk unaided. She was non-verbal, exhibited repetitive behaviours, displayed aggression towards others, had generalised muscular hypotonia, was incontinent and had chronic diarrhoea of unknown aetiology. She attended a special school where teachers have reported convulsive episodes but an EEG could not yet be performed. Body measurements could not be taken due to her defensive behaviour, but she did not appear to be small, dystrophic or microcephalic. Basic genetic testing, that is, karyotyping and array comparative genomic hybridisation (CGH), was performed due to ID of unclear aetiology and was unremarkable.

#### Family B

Patient 3 (female) was born at 38 weeks of gestation to reportedly unrelated parents originating from two neighbouring towns in Northwest Germany. An older sibling was healthy. Pregnancy and early postnatal adaptation were normal. Brainstem Electric Response Audiometry (BERA) was unremarkable. At the age of 15 months, she presented with generalised muscular hypotonia and motor delay. In the second year of life, a delay in speech development also became apparent. At 2 years and 3 months, she began to walk unaided. Cognitive assessment (SON-R 2½−7) at 4 years of age revealed mild ID (IQ 60), which was later confirmed using the WPPSI-III test. Multiple wake and sleep EEGs demonstrated pathological findings, including slow background activity with superimposed high-amplitude delta waves and photosensitivity, although no overt seizures were observed clinically. A tendency to constipation developed at the age of 6 years. Overall, she made continuous psychomotor developmental progress and was reported to communicate in short phrases. At her last clinical evaluation at 15 years of age, her physical measurements were normal: height 173 cm (+1.1 SD), weight 61.7 kg (+0.54 SD) and occipitofrontal head circumference 54.7 cm (−0.13 SD). Her facial gestalt (high frontal hairline, upward slanting palpebral fissures, strabismus, long columella with a short appearing philtrum and a thin upper lip, figure 1A) resembled those of the affected siblings of family 1. She was reported to be shy and anxious, and to exhibit behavioural problems in stressful situations.

Array CGH conducted at 3 years of age identified a small duplication of 1.49–3.03 kb at 11p15.5 encompassing *H19*, which was classified unlikely to be causative for the proband’s phenotype and not further segregated. ES performed at 10 years of age failed to identify pathogenic or likely pathogenic variants in established disease genes likely explaining the clinical phenotype.

### *PREP* variant identification and functional characterisation

#### Variant 1 (family A)

Trio GS revealed a homozygous frameshift variant in *PREP* in patient 1, predicted to result in a premature termination of translation: NM_002726.5:c.1570_1573dupCAAA, NC_000006.11:g.105730440_105730443dup, NP_002717.3:p.(Asn525Thrfs*5) (figure 1B). The variant is located in exon 13 of 15 and nonsense-mediated decay (NMD) of the aberrant mRNA is predicted (figure 1C). Both parents were heterozygous carriers. Sanger sequencing confirmed the variant in homozygous state in the affected sister (patient 2, online supplemental figure 1A), while five unaffected siblings were not homozygous (data not shown, figure 1D). The variant is listed four times in heterozygous state in gnomAD V4.1.1. Overall, no homozygous LoF *PREP* variants are displayed in the gnomAD database.^26^ No other convincing candidate variants were detected.

**Figure 2:**
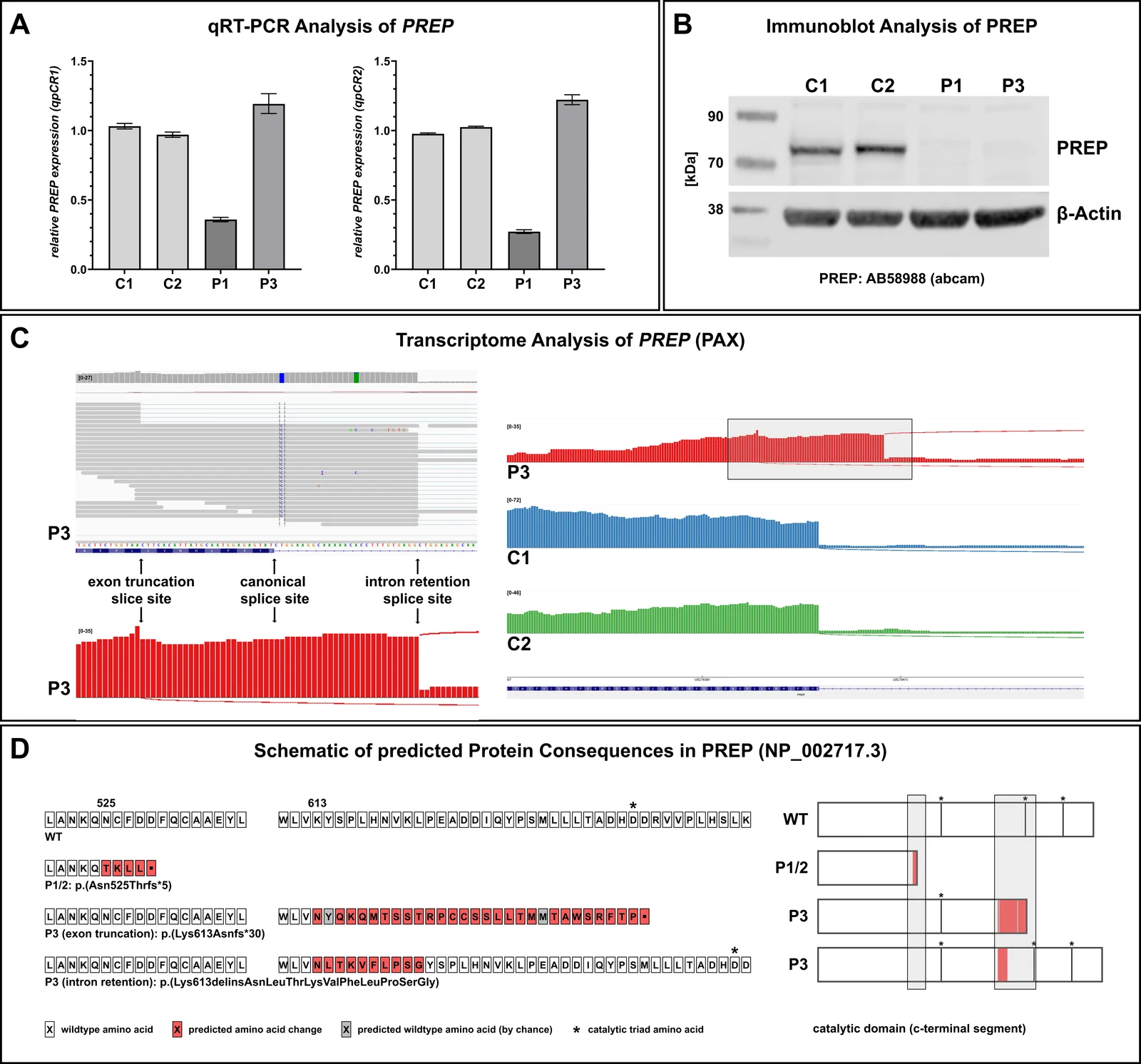
(A) qRT-PCR analysis of *PREP* in patient-derived lymphoblastoid cell line (LCL) cells showing relative *PREP* expression levels compared with two LCL controls using two different primer pairs. (B) Immunoblot detection of PREP in whole cell protein lysates of LCL cells using anti-PREP antibody ab58988. (C) Integrative Genomics Viewer screenshot and sashimi plot of RNA sequencing data showing aberrant splicing at the last *PREP* mRNA exon-intron boundary in peripheral blood sample from patient 3 compared with two peripheral blood samples from an inhouse database. (D) Schematic visualisation of the predicted consequences of identified PREP variants at the protein level compared with wildtype (WT).

To test if this variant has an impact on *PREP* expression, we performed qRT-PCR using LCL cells derived from patient 1. This showed a reduction in *PREP* mRNA levels to 25%–35% of normal compared with two control samples (figure 2A). Immunoblots using two different antibodies showed absence of PREP protein, thereby confirming that the variant is subject to NMD resulting in PREP deficiency (figure 2B, online supplemental figure 1F).

#### Variant 2 (family B)

Re-analysis of the exome data of patient 3 identified a homozygous splice variant in the last intron of *PREP*: NM_002726.5:c.1839-2A>G, NC_000006.11:g.105726315T>C (figure 1B,C). Sanger sequencing confirmed both parents as heterozygous carriers and the unaffected sibling not harbouring this variant in homozygous state (figure 1D, online supplemental figure 1A).

SpliceAI predicted the loss of the canonical acceptor site of exon 15 (Δ score: −0.99). Beyond this canonical acceptor loss, Pangolin also predicted the generation of two cryptic acceptor sites: (1) an intronic acceptor gain, 27 bp upstream of the canonical site (Δ score 0.41) and (2) an exonic acceptor gain, 25 bp downstream (Δ score 0.3) (online supplemental figure 1B).

To functionally investigate these predictions, RNA sequencing from a PAXgene-preserved blood sample was conducted. All detected *PREP* transcripts confirmed the loss of the canonical acceptor splice site resulting in two aberrant transcripts (figure 2C).

1. In 77% of all reads, the Pangolin-predicted intronic cryptic splice acceptor site was used, leading to a partial in-frame retention of intron 14: r.1838_1839ins[1839–27_1839-3;gg], p.(Lys613delinsAsnLeuThrLysValPheLeuProSerGly).
2. In the remaining 23% of reads, the Pangolin-predicted exonic cryptic acceptor site was used, leading to a partial skipping of exon 15, thereby causing a frameshift and the generation of the premature termination codon (PTC) r.1839_1863del, p.(Lys613Asnfs*30). Since this PTC is located in the last exon, this aberrant transcript is predicted to escape NMD.

We confirmed these results via qRT-PCR using aberrant isoform-specific primers and transcriptome sequencing from LCL-derived RNA (online supplemental figure 1C,D). To measure the impact of the aberrant splicing on *PREP* gene expression, qRT-PCR was performed, which showed no downregulation of *PREP* (figure 2A). Also, the PAX-derived transcriptome data revealed no significant alteration of *PREP* expression in comparison with the in-house control dataset (9.03 transcripts per million vs 12.02, p=0.586).

However, both aberrant isoforms are predicted to alter the C-terminal region of the protein, which could decrease the stability of the protein (AlphaFold predictions of isoform structure in online supplemental figure 1E):

The first aberrant in-frame isoform r.1838_1839ins, p.(Lys613delinsAsnLeuThrLysValPheLeuProSerGly) deletes the p.Lys613 residue and extends the hinge region due to the insertion of 10 aa (figure 2D).

The second aberrant isoform r.1839_1863del, p.(Lys613Asnfs*30) would alter the structure of the hydrolase domain by (a) deleting eight conserved residues p.Tyr614_Lys621, (b) altering p.Lys613 and the following 29 residues, including p.Asp641 of the catalytic triad and (c) deleting the last 67 C-terminal residues including p.His680 of the catalytic triad (figure 2D).

To test if a modified but stable PREP protein was expressed, we performed immunoblots, which revealed absence of PREP protein (figure 2B, online supplemental figure 1F). Importantly, the epitopes recognised by both antibodies are not located within the C-terminus (personal communication).

#### Variant 3 (family from literature)

Searching the SysNDD database, we found *PREP* listed as a candidate NDD/ID gene, based on the homozygosity of a frameshift variant in two siblings with global developmental delay, moderate ID and borderline microcephaly: NM_002726.5:c.1644_1647del, NC_000006.11:g.105730362_105730365del, NP_002717.3:p.Thr549Leufs*10.^9^

We classified variants 1 and 2 according to the American College of Medical Genetics and Genomics (ACMG) criteria and ClinGen recommendations as pathogenic under the assumption that *PREP*-related ID/NDD is inherited in an autosomal recessive mode and that biallelic LoF is the underlying pathomechanism.^27 28^ Further patient reports are needed to confirm this assumption.

1. NM_002726.5:c.1570_1573dup: PVS1, PM2_sup, PM3_sup, PP1_mod.
2. NM_002726.5:c.1839-2A>G: PVS1, PM2_sup, PM3_sup.

Variants were uploaded to the ClinVar database (SCV007124561 and SCV007124563).

## Discussion

In this report, we describe three patients from two unrelated families with biallelic LoF variants in *PREP* with a syndromic ID phenotype. In addition to moderate ID, all three had behavioural abnormalities in the form of repetitive, anxious or aggressive behaviour, strabismus, generalised muscular hypotonia and speech disturbance. Epilepsy was present in two of three patients. The diagnosis of DEE was made in one patient, seizures were reported in a further patient but diagnostics and treatment were refused and the EEG was pathologic with frequent epileptic discharges but not observed seizures in a third patient. All patients shared dysmorphic facial features, such as a high hairline, upslanted palpebral fissures, a low-hanging columella and a thin upper lip. Furthermore, urological and gastrointestinal symptoms, such as obstipation, diarrhoea and incontinence were noted in our patients. Overall, these comorbidities are among the most common in NDD.^29^ The clinical data of the siblings with the homozygous nonsense variant from the literature are scarce. Moderate ID was also reported in both, but no information regarding epilepsy was presented. In contrast, no microcephaly was observed in our patients.

NDD/ID is one of the most prevalent reasons for ES or GS in paediatrics. However, this currently only allows a diagnosis in about half of patients, with the diagnostic yield varying depending on the cohort and inclusion criteria.^30^ One explanation for this diagnostic gap is that only a quarter of the approximately 20 000 known protein coding genes has been associated with monogenic diseases (as of OMIM, November 2025), while several thousand disease-causing genes have still not been identified or published.^31 32^ In addition, thousands of genes are listed as candidate genes, but the published evidence is often insufficient to classify them as definite gene-disease associations, since they are either based on individual case reports with limited phenotypic information or no further functional characterisation has been conducted. Under these circumstances, however, the genes cannot be included in virtual panels by diagnostic laboratories and, consequently, no further cases outside the research setting can be found or adequately evaluated. Likewise, *PREP* was listed as a candidate disease gene in the supplementary data of a large cohort study.^9^

Through our analyses, that is, segregation within the unaffected siblings, RNA analysis and immunoblot analysis, we propose a pathogenic classification according to the ACMG/ACGS criteria.^27^ We recognise the critical importance of establishing highly valid gene-disease relationships. Thus, additional reports will be necessary to confirm our findings. The frameshift variant c.1570_1573dup, p.(Asn525Thrfs*5) in family A is likely subject to NMD, leading to reduced gene expression and absent protein expression. The intronic variant c.1839-2A>G in family B causes aberrant splicing, resulting in two aberrant isoforms. While one isoform is characterised by out-of-frame partial exon skipping and the formation of a premature stop codon in the last exon, the second aberrant isoform resulted in an in-frame insertion in the catalytic domain through partial intron retention. Both aberrant transcripts are predicted to escape NMD but would alter the C-terminal structure of the protein. While we could not detect any significant impact on *PREP* mRNA expression, no PREP protein was detectable using two different antibodies, suggesting that both isoforms are unstable and thus degraded. Since the homozygous frameshift variant c.1644_1647del, p.(Thr549Leufs*10) mentioned in the literature leads to a PTC in the same exon as the variant from family A, we assume that the variant is also subject to NMD and hence no stable protein is expressed. Our study emphasises how important it is to functionally validate variants and illustrates the difficulty to recruit further cases in the field of ultra-rare diseases, since despite a GeneMatcher submission, the second family of this study was only identified through personal communication with another institute. Furthermore, this report shows how important it is to re-analyse existing exome data, as in principle both cases could have been solved by exome analysis. The benefit of re-evaluating NGS data with respect to the diagnostic yield has been pointed out several times in recent years.^33^ Recently, by using the Talos tool that offers automated re-analysis, an additional diagnostic yield of approximately 5% in an unselected cohort was reported. Nearly one-third was explained by new gene-disease associations, which was even higher in the NDD subgroup, where almost 80% of diagnoses could be explained by novel gene-disease associations and newly detected CNVs.^34^

Even though our data suggest PREP deficiency as the underlying cause, the pathophysiological consequences remain unclear. After its discovery in 1971,^35^ PREP was initially considered as an extracellular enzyme implicated in neuropeptide turnover.^36^ Other studies demonstrated that PREP is active intracellularly in adult neurons and also expressed during neural differentiation.^37 38^ Because of its proline-specific peptidase activity, PREP gained considerable interest as a pharmacological target, especially in the field of neurocognitive disorders.^39^ However, there is currently no consensus on the substrates of PREP.^40^ In addition, it is increasingly assumed that PREP has multiple regulatory functions depending on its protein-protein-interactions (PPIs), which are presumably mediated via the non-enzymatic domain.^40^ In this regard, PREP was implicated in protein secretion, axonal transport and neural plasticity via direct interactions with L-tubulin^41 42^ and growth-associated protein 43.^43^ Furthermore, the conformation of PREP and the environment it is expressed in are considered important for PREP function.^15 44 45^ However, the precise physiological function of PREP remains largely elusive.^40^

In this respect, we hope that our study will spark a series of follow-up investigations in the field of PREP-related pathophysiology.

Although no human PREP deficiency has been described thus far, its importance for neural development can be inferred from the *Prep*-KO mice, where disturbed cone growth and synaptic function were observed.^43^ Other studies in *Prep*-KO mice showed an association between PREP and neuroplasticity modulation, probably mediated by an inflammatory response in which polysialylated neural cell adhesion molecule levels are altered in the brain.^10^

Noteworthy, PREP has one homologous protein named PREPL, which lacks protease activity and was assigned to function as a thioesterase.^46 47^ The intracellular localisations of PREP and PREPL are similar^48^ and it has been speculated that the PPI interactions of PREPL may be comparable to those of PREP.^49^ Several patients with biallelic LoF variants in PREPL have been described with a congenital myasthenic syndrome with neonatal hypotonia and growth failure (CMS22, MIM: 616224). Interestingly, patients often had moderate ID, a feature that was consistently observed also in our patients.

In conclusion, our data indicate PREP deficiency as the underlying cause of a syndromic ID disorder, which we would name ‘PREP-related NDD’ according to the recommended dyadic nomenclature.^50^ In total, five individuals with three different LoF variants have been described so far and we suggest adding *PREP* to virtual ID/NDD and epilepsy panels to identify additional patients and validate the proposed gene-disease association.

## Supplementary material

10.1136/jmg-2026-111501 online supplemental file 1

## Data Availability

All data relevant to the study are included in the article or uploaded as supplementary information.

## References

[1] American Psychiatric Association (2022). Diagnostic and statistical manual of mental disorders.

[2] World Health Organization, United Nations Children’s Fund (2023). Global report on children with developmental disabilities: from the margins to the mainstream.

[3] Francés L, Quintero J, Fernández A (2022). Current state of knowledge on the prevalence of neurodevelopmental disorders in childhood according to the DSM-5: a systematic review in accordance with the PRISMA criteria. Child Adolesc Psychiatry Ment Health.

[4] SysNDD https://sysndd.dbmr.unibe.ch/.

[5] Manickam K, McClain MR, Demmer LA (2021). Exome and genome sequencing for pediatric patients with congenital anomalies or intellectual disability: an evidence-based clinical guideline of the American College of Medical Genetics and Genomics (ACMG). Genet Med.

[6] van der Sanden BPGH, Schobers G, Corominas Galbany J (2023). The performance of genome sequencing as a first-tier test for neurodevelopmental disorders. Eur J Hum Genet.

[7] Hamanaka K, Fujita A, Miyatake S (2025). Genome sequencing provides high diagnostic yield and new etiological insights for intellectual disability and developmental delay. NPJ Genom Med.

[8] Chong JX, Berger SI, Baxter S (2024). Considerations for reporting variants in novel candidate genes identified during clinical genomic testing. Genet Med.

[9] Hu H, Kahrizi K, Musante L (2019). Genetics of intellectual disability in consanguineous families. Mol Psychiatry.

[10] Höfling C, Kulesskaya N, Jaako K (2016). Deficiency of prolyl oligopeptidase in mice disturbs synaptic plasticity and reduces anxiety-like behaviour, body weight, and brain volume. Eur Neuropsychopharmacol.

[11] EC 3.4.21.26. https://iubmb.qmul.ac.uk/enzyme/EC3/4/21/26.html.

[12] Myöhänen TT, García-Horsman JA, Tenorio-Laranga J (2009). Issues about the physiological functions of prolyl oligopeptidase based on its discordant spatial association with substrates and inconsistencies among mRNA, protein levels, and enzymatic activity. J Histochem Cytochem.

[13] Fülöp V, Böcskei Z, Polgár L (1998). Prolyl oligopeptidase: an unusual beta-propeller domain regulates proteolysis. Cell.

[14] Tsirigotaki A, Elzen RV, Veken PVD (2017). Dynamics and ligand-induced conformational changes in human prolyl oligopeptidase analyzed by hydrogen/deuterium exchange mass spectrometry. Sci Rep.

[15] Männistö PT, García-Horsman JA (2017). Mechanism of Action of Prolyl Oligopeptidase (PREP) in Degenerative Brain Diseases: Has Peptidase Activity Only a Modulatory Role on the Interactions of PREP with Proteins?. Front Aging Neurosci.

[16] Holtgrewe M, Stolpe O, Nieminen M (2020). VarFish: comprehensive DNA variant analysis for diagnostics and research. Nucleic Acids Res.

[17] Falb RJ, Müller AJ, Klein W (2023). Bi-allelic loss-of-function variants in *KIF21A* cause severe fetal akinesia with arthrogryposis multiplex. J Med Genet.

[18] Park J, Tucci A, Cipriani V (2022). Heterozygous UCHL1 loss-of-function variants cause a neurodegenerative disorder with spasticity, ataxia, neuropathy, and optic atrophy. Genet Med.

[19] https://github.com/imgag/megSA.

[20] Jaganathan K, Kyriazopoulou Panagiotopoulou S, McRae JF (2019). Predicting Splicing from Primary Sequence with Deep Learning. Cell.

[21] Zeng T, Li YI (2022). Predicting RNA splicing from DNA sequence using Pangolin. Genome Biol.

[22] Sobreira N, Schiettecatte F, Valle D (2015). GeneMatcher: a matching tool for connecting investigators with an interest in the same gene. Hum Mutat.

[23] Weisschuh N, Mazzola P, Zuleger T (2024). Diagnostic genome sequencing improves diagnostic yield: a prospective single-centre study in 1000 patients with inherited eye diseases. J Med Genet.

[24] GitHub GitHub - imgag/megSAP: a medical genetics sequence analysis pipeline. https://github.com/imgag/megSAP.

[25] Kopp J, Koch LA, Lyubenova H (2024). Loss-of-function variants affecting the STAGA complex component SUPT7L cause a developmental disorder with generalized lipodystrophy. Hum Genet.

[26] Chen S, Francioli LC, Goodrich JK (2024). A genomic mutational constraint map using variation in 76,156 human genomes. Nature New Biol.

[27] Richards S, Aziz N, Bale S (2015). Standards and guidelines for the interpretation of sequence variants: a joint consensus recommendation of the American College of Medical Genetics and Genomics and the Association for Molecular Pathology. Genet Med.

[28] Clinical Genome Resource ClinGen variant classification guidance - ClinGen. https://clinicalgenome.org/tools/clingen-variant-classification-guidance/.

[29] Dingemans AJM, Jansen S, van Reeuwijk J (2024). Prevalence of comorbidities in individuals with neurodevelopmental disorders from the aggregated phenomics data of 51,227 pediatric individuals. Nat Med.

[30] Wigby KM, Brockman D, Costain G (2024). Evidence review and considerations for use of first line genome sequencing to diagnose rare genetic disorders. NPJ Genom Med.

[31] Home - OMIM. www.omim.org.

[32] Antonarakis SE (2019). Carrier screening for recessive disorders. Nat Rev Genet.

[33] Dai P, Honda A, Ewans L (2022). Recommendations for next generation sequencing data reanalysis of unsolved cases with suspected Mendelian disorders: A systematic review and meta-analysis. Genet Med.

[34] Welland MJ, Ahlquist KD, De Fazio P (2025). Scalable automated reanalysis of genomic data in research and clinical rare disease cohorts. medRxiv.

[35] Walter R, Shlank H, Glass JD (1971). Leucylglycinamide released from oxytocin by human uterine enzyme. Science.

[36] García-Horsman JA, Männistö PT, Venäläinen JI (2007). On the role of prolyl oligopeptidase in health and disease. Neuropeptides.

[37] Moreno-Baylach MJ, Felipo V, Männistö PT (2008). Expression and traffic of cellular prolyl oligopeptidase are regulated during cerebellar granule cell differentiation, maturation, and aging. Neuroscience.

[38] Hannula MJ, Männistö PT, Myöhänen TT (2011). Sequential expression, activity and nuclear localization of prolyl oligopeptidase protein in the developing rat brain. Dev Neurosci.

[39] Männisto PT, Venäläinen J, Jalkanen A (2007). Prolyl oligopeptidase: a potential target for the treatment of cognitive disorders. Drug News Perspect.

[40] García-Horsman JA (2020). The role of prolyl oligopeptidase, understanding the puzzle. Ann Transl Med.

[41] Roßner S, Schulz I, Zeitschel U (2005). Brain Prolyl Endopeptidase Expression in Aging, APP Transgenic Mice and Alzheimer’s Disease. Neurochem Res.

[42] Schulz I, Zeitschel U, Rudolph T (2005). Subcellular localization suggests novel functions for prolyl endopeptidase in protein secretion. J Neurochem.

[43] Di Daniel E, Glover CP, Grot E (2009). Prolyl oligopeptidase binds to GAP-43 and functions without its peptidase activity. Mol Cell Neurosci.

[44] Szeltner Z, Juhász T, Szamosi I (2013). The loops facing the active site of prolyl oligopeptidase are crucial components in substrate gating and specificity. Biochim Biophys Acta.

[45] Szeltner Z, Morawski M, Juhász T (2010). GAP43 shows partial co-localisation but no strong physical interaction with prolyl oligopeptidase. Biochimica et Biophysica Acta (BBA) - Proteins and Proteomics.

[46] Szeltner Z, Alshafee I, Juhász T (2005). The PREPL A protein, a new member of the prolyl oligopeptidase family, lacking catalytic activity. Cell Mol Life Sci.

[47] Martens K, Derua R, Meulemans S (2006). PREPL: a putative novel oligopeptidase propelled into the limelight. Biol Chem.

[48] Morawski M, Nuytens K, Juhasz T (2013). Cellular and ultra structural evidence for cytoskeletal localization of prolyl endopeptidase-like protein in neurons. Neuroscience.

[49] Boonen K, Régal L, Jaeken J (2011). PREPL, a prolyl endopeptidase-like enzyme by name only?--Lessons from patients. CNS Neurol Disord Drug Targets.

[50] Thaxton C, Biesecker LG, DiStefano M (2024). Implementation of a dyadic nomenclature for monogenic diseases. Am J Hum Genet.

