# Supplementary material for: Identification of biallelic loss-of-function PREP variants in three individuals with syndromic intellectual disability
Source: J Med Genet. 2026 Jun 26;63(9):e111501. doi: 10.1136/jmg-2026-111501 (PMC13539837; doi:10.1136/jmg-2026-111501)
Supplement: Supplementary file 1 [file jmg-63-9-s001.docx]

**Supplemental Material**

**Table 1 (**Primer list):

| NAME | SEQUENCE | POSITION* |
| --- | --- | --- |
| qPCR1 FWD | TGTAGGGTACAGCGGTCAGA | 105776771-105776752 |
| qPCR1 REV | CTCTTGGCTCCAGCTCCTCT | 105771596-105771615 |
| qPCR2 FWD | CCACCCTTCAGTACATCGTGG | 105726191-105726171 |
| qPCR2 REV | TCCTCTATCACTTTGGCTGTGG | 105726077-105726098 |
| qPCR_ET FWD | TGGAGTAATGGACATGCTGAAGT | 105729725-105729703 |
| qPCR_ET REV | GCTTCTGGTAATTGACAAGCCA | 105726278-105726288  105729621-105729631 |
| qPCR_IR FWD | GGCTTGTCAACCTCACAAAGG | 105729630-105729621  105726340-105726330 |
| qPCR_IR REV | CATGGTCAGCAGTGAGGAGC | 105726231-105726250 |
| GAPDH FWD | CTGCACCACCAACTGCTTAG | 6646484-6646503 |
| GAPDH REV | ACAGTCTTCTGGGTGGCAGT | 6646787-6646768 |
|  |  | **PREP*: NC_000006.11:g.X  *GAPDH*:  NC_000012.11:g.X |

**Supplementary Figure 1:**

A: Segregation analysis of identified *PREP* variants in family A and B.

B: Visualizations of SpliceAI and Pangolin splicing predictions (derived from https://spliceailookup.broadinstitute.org) aligned with IGV screenshot of RNA sequencing data from patient 3.

C: qRT-PCR analysis of *PREP* in patient-derived LCL cells confirming aberrant splicing in patient 3 using aberrant isoform specific primers.

D: IGV screenshot and sashimi plot of RNA sequencing data confirming aberrant splicing patterns in LCL cells from patient 3.

E: Schematic of AlphaFold predictions for wild type and aberrant PREP isoforms in patient 3 (derived from <https://alphafold.ebi.ac.uk>). p.(Lys613delinsXaa[10]) represents p.(Lys613delinsAsnLeuThrLysValPheLeuProSerGly).

F: Confirmatory immunoblot detection of PREP in whole cell protein lysates of LCL cells using anti-PREP antibody ab246978. Artificial band at 70 kDa, as documented in the manufacturer’s sample.
